# Activating Transcription Factor 4 Promotes Angiogenesis of Breast Cancer through Enhanced Macrophage Recruitment

**DOI:** 10.1155/2015/974615

**Published:** 2015-03-25

**Authors:** Chen Liu, Zongjin Li, Lina Wang, Lingling Tong, Ningning He, Yanan Chen, Yanhua Liu, Zhongjun Wu, Peiqing Sun, Rong Xiang, Guosheng Ren, Weijun Su

**Affiliations:** ^1^The First Affiliated Hospital of Chongqing Medical University, Chongqing 400016, China; ^2^School of Medicine, Nankai University, Tianjin 300071, China; ^3^State Key Lab of Experimental Hematology, Institute of Hematology and Blood Diseases Hospital, Chinese Academy of Medical Sciences, Tianjin 300052, China; ^4^Department of Clinical Laboratory, Xiamen International Travel Healthcare Center, Xiamen 361012, China; ^5^Department of Cell and Molecular Biology, The Scripps Research Institute, La Jolla, CA 92037, USA

## Abstract

Angiogenesis plays an important role in the progression of tumor. Besides being regulated by tumor cells per se, tumor angiogenesis is also influenced by stromal cells in tumor microenvironment (TME), for example, tumor associated macrophages (TAMs). Activating transcription factor 4 (ATF4), a member of the ATF/CREB family, has been reported to be related to tumor angiogenesis. In this study, we found that exogenous overexpression of ATF4 in mouse breast cancer cells promotes tumor growth via increasing tumor microvascular density. However, ATF4 overexpression failed to increase the expression level of a series of proangiogenic factors including vascular endothelial growth factor A (VEGFA) in tumor cells in this model. Thus, we further investigated the infiltration of proangiogenic macrophages in tumor tissues and found that ATF4-overexpressing tumors could recruit more macrophages via secretion of macrophage colony stimulating factor (M-CSF). Overall, we concluded that exogenous overexpression of ATF4 in breast cancer cells may facilitate the recruitment of macrophages into tumor tissues and promote tumor angiogenesis and tumor growth indirectly.

## 1. Introduction

Angiogenesis plays an important role in the progression of tumor. Insufficient oxygen supply to meet the demands of proliferating cancer cells gives rise to hypoxic tumor microenvironment (TME). And oxygen-deprived cancer cells respond to the hypoxic stress by inducing a series of cellular responses leading to angiogenesis [1–4]. Besides being regulated by tumor cells per se, it has been found that stromal cells in TME also play significant roles in tumor angiogenesis [5–8]. Among them, tumor associated macrophages (TAMs) are most intensively studied. Via either producing proangiogenic factors or physically assisting angiogenic sprouting, TAMs have been found to promote tumor angiogenesis directly or indirectly [8–11].

Activating transcription factor 4 (ATF4) belongs to the ATF/CREB family. It has been found that ATF4 expression is increased in response to tumor microenvironment stresses including oxygen deprivation [12–15], and it takes part in the adaptation to hypoxia [16, 17]. In previous studies, ATF4 was shown to mediate the VEGF-dependent tumor growth and angiogenesis triggered by osteopontin (OPN) [18]. However, it has not been discussed before whether exogenous overexpression of ATF4 in tumor cells can also influence tumor angiogenesis via regulating stromal cells in TME.

In this study, we overexpressed ATF4 in mouse breast cancer 4T1 and 4TO7 cells. In these two models, ATF4 overexpression facilitates the macrophage infiltration into breast cancer tissues via enhanced secretion of M-CSF and thus promotes tumor angiogenesis and tumor growth indirectly via recruiting proangiogenic macrophages.

## 2. Materials and Methods

### 2.1. Cell Culture

Mouse breast cancer cell lines 4T1 and 4TO7 and mouse macrophage cell line Raw264.7 were cultured with 1640 medium (ThermoFisher Scientific, Hudson, NH) supplemented with 10% fetal bovine serum (FBS) (Corning, Lowell, MA) and 1% penicillin-streptomycin solution (Gibco, Rockville, MD). All cell lines were maintained at 37°C in a 5% CO2 incubator. To be tracked* in vivo*, 4T1 cells were transduced with a self-inactivating lentiviral vector carrying a ubiquitin promoter driving firefly luciferase reporter gene to obtain the 4T1-Luc cells.

### 2.2. Plasmid Constructs

The lentivirus system including three packaging vectors (pCMV-VSVG, pRRE, and pRSV-REV) and the expression vector (pLV-EF1*α*-MCS-IRES-Bsd) were purchased from Biosettia Company (San Diego, CA, USA). Full-length mouse ATF4 was cloned from 4T1 cell cDNA, and the PCR-amplified fragment was inserted into the MCS of pLV-EF1*α*-MCS-IRES-Bsd vector to obtain the pLV-ATF4-Bsd plasmid.

### 2.3. Lentivirus Packaging and Generation of Stable Transfectants

293T cells were transfected with the lentivirus packaging vectors and pLV-ATF4-Bsd plasmid using Lipo-2000 (Invitrogen, Carlsbad, CA) according to the manufacturer's instructions. 40 hours after transfection, the supernatant was collected and stored at −80°C until use.

4T1-Luc or 4TO7 cells were seeded into 6-well plates at a density of 10^5^ per well. 24 hours later, the medium was changed with the mixture of 2 mL complete medium and 1 mL lentivirus supernatant, and the cells were centrifuged at 1600 rpm for 1 h at 37°C. After centrifugation, the lentivirus was discarded, and the tumor cells were cultured for another 48 h before the selection medium with Bsd (Sigma, St. Louis, MO) was added. Four days later, the protein of the selected 4T1-Luc-ATF4 or 4TO7-ATF4 cells was collected to detect the ATF4 expression level.

### 2.4. Cell Counting Kit-8 (CCK-8) Assay

CCK-8 assay kit was bought from Dojindo Molecular Technologies (Kumamoto, Japan). Cells for assay were cultured in 96-well plates, and the culture medium was changed with 100 *μ*L fresh medium per well before each measurement. 10 *μ*L CCK-8 working solution was added to each well, and the cells were incubated for another 2 hours. Two hours later, the absorbance of the samples at 450 nm was measured using GloMax-Multi Detection System (Promega, Madison, WI).

### 2.5. Western Blot Analysis

Total cell protein was prepared by lysis of cells with the radioimmunoprecipitation assay (RIPA) buffer, and the protein concentrations were determined by the bicinchoninic acid (BCA) protein assay kit (ThermoFisher Scientific). Proteins were examined with specific antibodies against ATF4 (1 : 1000 dilution, Santa Cruz Biotechnology, Santa Cruz, CA), luciferase (1 : 1000 dilution, Promega), Ki67 (1 : 1000 dilution, Abcam, Cambridge, MA), M-CSF (1 : 1000 dilution, Santa Cruz Biotechnology), and *β*-actin (1 : 5000 dilution, Santa Cruz Biotechnology), followed by peroxidase-conjugated secondary antibodies (Abcam). The reactions were detected using Immobilon Western Chemiluminescent HRP Substrate (Millipore, Billerica, MA).

### 2.6. Real-Time PCR

Total RNA was extracted using TRIzol reagent (Invitrogen) and reverse transcribed with PrimeScript RT reagent (TaKaRa, Shiga, Japan). SYBR Premix Ex Taq (TaKaRa) was used to amplify cDNA for real-time PCR. The primers used for real-time PCR are as follows: mouse hypoxia inducible factor 1 alpha (mHIF-1alpha) (5′-GTGCACCCTAACAAGCCGGGG-3′/5′-AGCACCAAGCACGTCATGGGT-3′), mouse vascular endothelial growth factor A (mVEGFA) (5′-AGGGCTATACTGCCCTCCAA-3′/5′-ACGCGAGTCTGTGTTTTTGC-3′), mouse placenta growth factor (mPlGF) (5′-CACTTGCTTCTTACAGGTCC-3′/5′-CACCTCATCAGGCTATTCAT-3′), mouse platelet derived growth factor, B polypeptide (mPDGF-B) (5′-TGTAATCGCCGAGTGCAAGA-3′/5′-CATTGCACATTGCGGTTATTG-3′), mouse angiopoietin 1 (mANG-1) (5′-AGCTACCAACAACAACAGCA-3′/5′-GCAAAGGCTGATAAGGTTATGA-3′), mouse angiopoietin 2 (mANG-2) (5′-AGCCACGGTCAACAACTCGC-3′/5′-TCTTCTTTACGGATAGCAAC-3′), mouse granulocyte colony stimulating factor (mG-CSF) (5′-CGTTCCCCTGGTCACTGTC-3′/5′-TAGAGCCTGCAGGAGACCTT-3′), mouse macrophage colony stimulating factor (mM-CSF) (5′-GCAACTCAGCCACCCCCGTT-3′/5′-AAAACGGGCCACAGGCCTGG-3′), and mouse granulocyte-macrophage colony stimulating factor (mGM-CSF) (5′-CTCCGGAAACGGACTGTGAA-3′/5′-AGGGCTATACTGCCCTCCAA-3′). Data analysis was performed using GelDoc XR (BioRad, Berkeley, CA, USA).

### 2.7. Transwell Migration Assay

1 × 10^5^ Raw264.7 cells in 200 *μ*L basic medium were seeded upon the 24-well Millicell Hanging Cell Culture Inserts (Millipore) with conditioned medium of ATF4 overexpressing tumor cells or control cells in the lower chamber. After incubation for 24 hours, the inserts were taken out, and the cells were removed from the upper chamber with a cotton swab. Cells which have migrated through the pores and attached to the reverse side of the membrane were fixed with 4% formaldehyde in PBS, followed by staining with 0.5% crystal violet for 20 min. Cells of 5 randomly chosen fields per membrane were counted under the microscope at 200x and statistically analyzed.

### 2.8. Animal Works

Six-week-old female Balb/c mice were purchased from the Experimental Animal Institute, Chinese Academy of Medical Sciences (Beijing, China). All experimental procedures were conducted in conformity with institutional guidelines for The Care and Use of Laboratory Animals in Nankai University, Tianjin, China, and conformed to the National Institutes of Health (NIH) Guide for Care and Use of Laboratory Animals. 4T1-Luc-ATF4 or 4T1-Luc-MCS cells (5 × 10^4^ per mouse) were injected subcutaneously to the mice. Bioluminescence imaging was performed on day 0, day 4, day 7, day 10, and day 13. The tumor volume was measured with microcalipers from day 8 to day 31. Mice were sacrificed on day 16 or day 31, and the tumor tissues were embedded within OCT and stored at −80°C until use.

### 2.9. Bioluminescence Imaging

Bioluminescence imaging of Fluc was performed using the IVIS Lumina II system (Xenogen, Alameda, CA). After intraperitoneal injection of D-Luciferin (150 mg/kg) (Invitrogen), each mouse was imaged for 1–5 minutes under anesthesia with isoflurane. Bioluminescence signals were quantified in units of maximum photons per second per cm square per steradian (photons/sec/cm^2^/sr).

### 2.10. Immunofluorescence Staining

For immunofluorescence staining, specific antibodies against F4/80 (1 : 50 dilution, Abcam) and mouse CD31 (1 : 100 dilution, BD Biosciences, Bedford, MA), followed by Alexa Fluor 594 labeled-secondary antibodies (Invitrogen), were used for detection. Then the sections were counterstained with 4′,6-diamidino-2-phenylindole (DAPI), mounted, and observed under the microscope.

### 2.11. Immunohistochemical Staining

Specific antibody against M-CSF (1 : 50 dilution, Santa Cruz Biotechnology) followed by peroxidase-conjugated goat anti-mouse IgG (Vector Laboratories, Burlingame, CA) was used. DAB Peroxidase Substrate Kit (Vector Laboratories) was employed for detection. Then the sections were costained with hematoxylin, hydrated, mounted, and observed under the microscope.

### 2.12. Statistical Analysis

Statistics were calculated using SigmaStat for Windows Version 3.5. For comparison between two groups, two-tailed Student's *t*-test was used. Differences were considered as significant at *P* values of less than 0.05.

## 3. Results and Discussion

### 3.1. ATF4 Promotes the Growth of Breast Cancer* In Vivo* without Influencing Breast Cancer Cell Proliferation* In Vitro*

First, to study the influence of ATF4 on the growth of breast cancer, we overexpressed ATF4 in mouse breast cancer 4T1-Luc cells and injected the 4T1-Luc-ATF4 cells subcutaneously to Balb/c mice. Western blots were used to confirm the overexpression of ATF4 (Figure S1(a), available online at http://dx.doi.org/10.1155/2014/974615). And the expression level of Luc was not influenced by ATF4 overexpression (Figure S1(b)).

**Figure 1:**
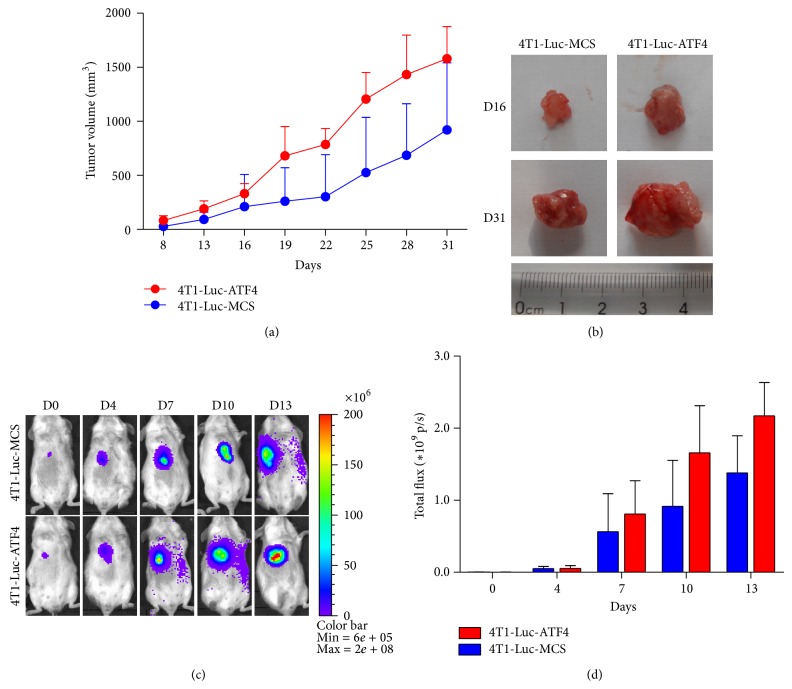
ATF4 promotes the growth of breast cancer* in vivo*. (a) ATF4-overexpressing breast cancer grew faster than MCS group* in vivo*. (b) Representative tumor tissues from ATF4 group and MCS group on day 16 and day 31. (c) Fluc imaging of subcutaneous breast cancers from ATF4 group and MCS group on day 0, day 4, day 7, day 10, and day 13. For each group, a representative mouse at each time point was shown. (d) Quantitative analysis of BLI signals in (c) was shown as photons/sec/cm^2^/sr. MCS is the abbreviation of multiple cloning sites, and MCS group is used as control here.

The growth rate of ATF4-overexpressing tumors was significantly enhanced compared to MCS tumors (used as control here), and the average volume of ATF4-overexpressing tumors was nearly twice that of MCS tumors on day 31 (Figure 1(a)).  Figure 1(b) shows the representative tumor tissues from ATF4 group and MCS group on day 16 and day 31. For it is not an exact method to measure the volume of tumors with microcalipers in early stage, we also employed bioluminescence imaging (BLI) to assess the growth rate of tumors. Via BLI, we found that even in early stage ATF4-overexpressing tumors grew with a much higher rate (Figures 1(c) and 1(d)).

**Figure 2:**
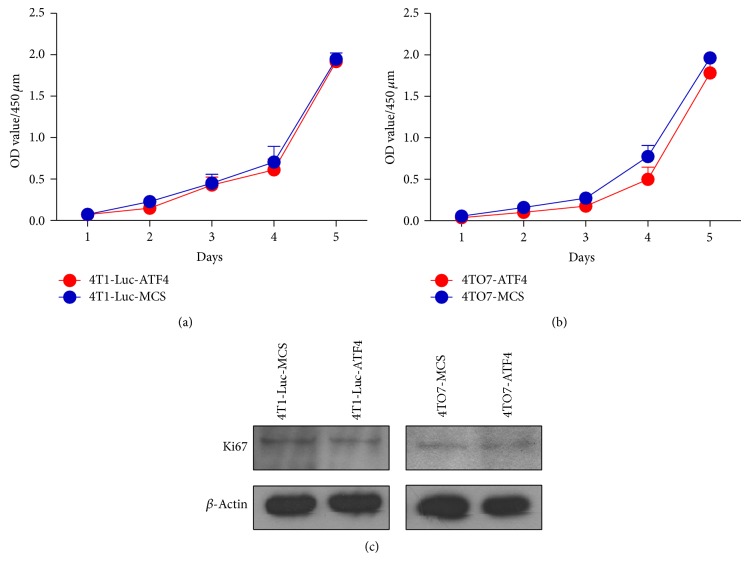
ATF4 does not influence the proliferation of mouse breast cancer cells* in vitro*. (a) ATF4 overexpression did not influence the proliferation of 4T1-Luc cells* in vitro*. (b) ATF4 overexpression did not influence the proliferation of 4TO7 cells* in vitro*. (c) ATF4 overexpression did not influence Ki67 expression level in 4T1-Luc and 4TO7 cells.

To investigate whether the promotion effect on breast cancer growth depends on the influence of ATF4 on cancer cell proliferation, CCK-8 cell proliferation assay was performed.  Figure 2(a) shows that ATF4 overexpression did not significantly influence the proliferation of 4T1-Luc cells* in vitro*. Similarly, overexpression of ATF4 in another mouse breast cancer cell line, 4TO7, also did not obviously influence its proliferation (Figures S1(a) and 2(b)). We also detected the expression of Ki67, a proliferation marker in ATF4-overexpressing breast cancer cells by western blots. In either 4T1-Luc cells or 4TO7 cells, ATF4 overexpression failed to enhance the Ki67 expression level (Figure 2(c)). Overall, these data demonstrated that ATF4 could not influence the proliferation of mouse breast cancer cells. Thus, we hypothesized that ATF4 may promote the growth of breast cancer* in vivo* through regulation of TME.

### 3.2. ATF4 Increases the Microvascular Density of Breast Cancer but Fails to Upregulate the Expression of Proangiogenic Factors in Breast Cancer Cells

**Figure 3:**
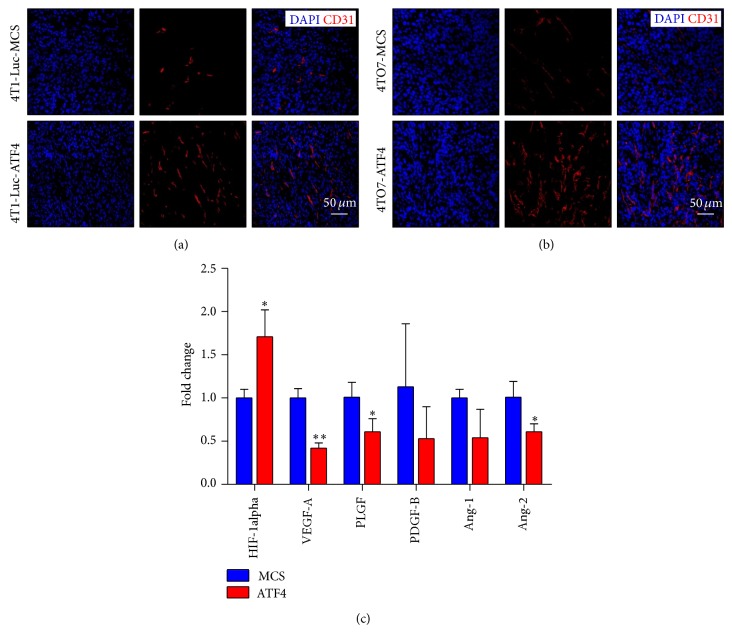
ATF4 increases the microvascular density in breast cancer tissue. (a) Immunofluorescence staining of CD31 showed the increased microvascular density in ATF4-overexpressing 4T1-Luc breast cancers. (b) Immunofluorescence staining of CD31 showed the increased microvascular density in ATF4-overexpressing 4TO7 breast cancers. (c) ATF4 did not significantly influence the expression of proangiogenic factors.

Angiogenesis is an important limiting factor on tumor growth. There have been reports that ATF4 relates to tumor angiogenesis. This leads us to investigate whether ATF4 overexpression can regulate breast cancer angiogenesis and also promote breast cancer growth* in vivo*. We sacrificed 4T1-Luc or 4TO7 tumor-bearing mice on day 31 and detected microvascular density in tumor tissues. Figures 3(a) and 3(b) show that the microvascular density of ATF4-overexpressing tumors is much higher than MCS groups in these two models.

For there have been reports that ATF4 can regulate the expression of VEGF [16–18], we initially speculated that maybe exogenous ATF4 overexpression can promote tumor angiogenesis via upregulation of proangiogenic factors in breast cancer cells. However, to our surprise, real-time PCR showed that ATF4 failed to enhance the mRNA level of most proangiogenic factors we detected in breast cancer cells. In fact, the expression level of VEGF-A, PlGF, PDGF-B, Ang-1, and Ang-2 even tended to decrease in ATF4-overexpressing breast cancer cells. The only exception was HIF-1alpha, whose expression level was upregulated by ATF4 less than two times (Figure 3(c)). Consistent with our results, it has been reported that ablation of ATF4 in mice leads to dramatic decrease of HIF-1alpha expression in osteoblasts [17].

### 3.3. ATF4 Facilitates the Recruitment of Macrophages via Enhanced Secretion of M-CSF

**Figure 4:**
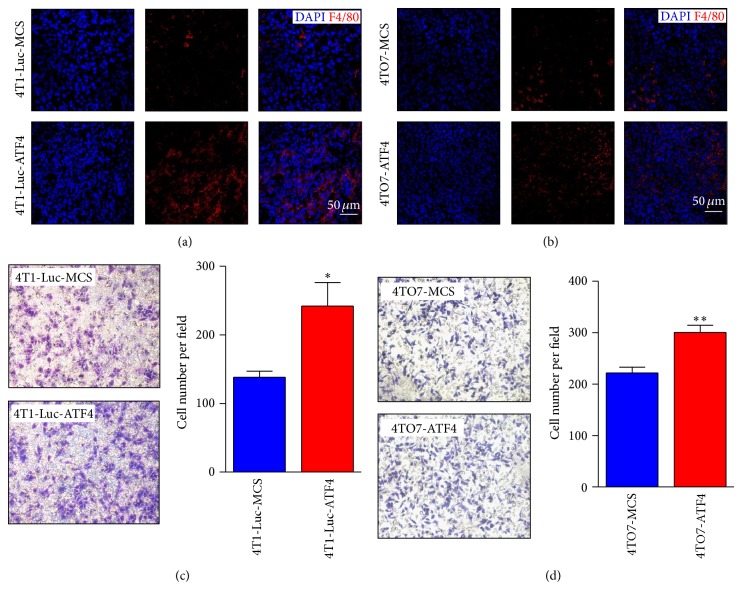
ATF4 facilitates the recruitment of macrophages both* in vitro* and* in vivo*. (a) Immunofluorescence staining of F4/80 showed more macrophages infiltrating into ATF4-overexpressing 4T1-Luc breast cancer tissue compared to MCS group. (b) Immunofluorescence staining of F4/80 showed more macrophages infiltrating into ATF4-overexpressing 4TO7 breast cancer tissue compared to MCS group. (c) More Raw264.7 cells were recruited by the conditioned medium of ATF4-overexpressing 4T1-Luc cells. Data were representative of three independent experiments. (d) More Raw264.7 cells were recruited by the conditioned medium of ATF4-overexpressing 4TO7 cells. Data were representative of three independent experiments.

Besides being regulated by tumor cells per se, tumor angiogenesis can also be influenced by stromal cells in TME. In previous studies, it has been reported that TAMs in TME are proangiogenic. Thus we detected whether the infiltration of macrophages in tumor tissues was influenced by ATF4 overexpression. Immunofluorescence staining for F4/80, a macrophage marker, showed that there were more macrophages recruited into ATF4-overexpressing breast cancer tissues in both 4T1-Luc and 4TO7 model (Figures 4(a) and 4(b)). And this phenomenon was further confirmed by transwell migration assay* in vitro*. Compared to MCS groups, more mouse macrophage Raw264.7 cells were recruited to the lower chamber by the conditioned medium of ATF4-overexpressing breast cancer cells (Figures 4(c) and 4(d)).

In most malignant tumors, macrophages compose the most prominent component of stromal cells in TME. Once infiltrated into tumor tissues, macrophages undergo profound changes in gene transcription, thus resulting in the proangiogenic “tumor associated macrophages (TAMs).” The overall TAMs number has been found to be correlated with increased microvascular density in a variety of tumors, for example, pulmonary adenocarcinoma [19], endometrial carcinoma [20], and also breast cancer [21]. And our results further confirmed this phenomenon in breast cancer.

**Figure 5:**
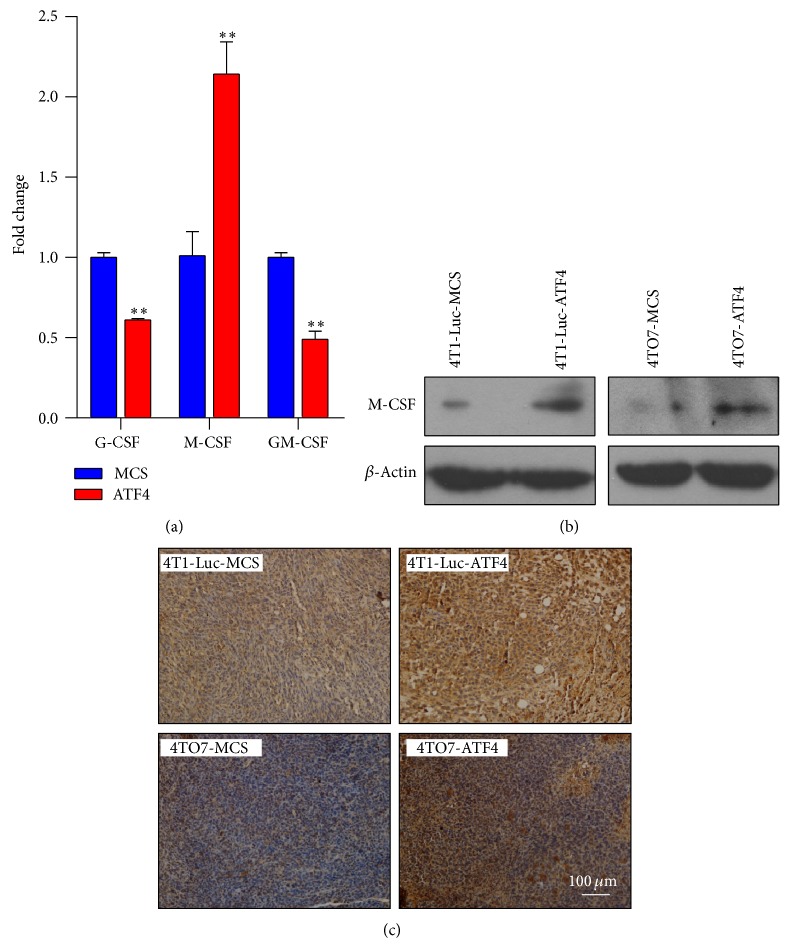
ATF4 increases the expression of M-CSF in breast cancer cells. (a) The mRNA level of M-CSF was increased in ATF4-overexpressing breast cancer cells. (b) The protein level of M-CSF was increased in ATF4-overexpressing breast cancer cells. (c) Immunohistochemical staining showed increased M-CSF level in ATF4-overexpressing breast cancer tissues.

Further, we wanted to uncover the soluble factors through which more macrophages were recruited to ATF4-overexpressing tumors. By real-time PCR, we found that among G-CSF, M-CSF, and GM-CSF, which have been reported to be related to the recruitment and development of macrophages, only M-CSF expression level was significantly elevated in ATF4-overexpressing breast cancer cells (Figure 5(a)), which was also confirmed in protein level by western blots (Figure 5(b)). Furthermore, immunohistochemical staining also demonstrated the elevated level of M-CSF in ATF4-overexpressing breast cancer tissues (Figure 5(c)).

A series of previous studies implicate M-CSF derived from breast cancer cells as a potent chemoattractant for macrophages [22–25]. There have been reports that ATF4 was largely modulated by M-CSF signaling and was also crucial for M-CSF induction of RANK expression in bone marrow monocytes [26]. However, there have been no reports whether M-CSF can also be regulated by ATF4 before. In our study, ectopic overexpression of ATF4 in breast cancer cells led to the enhanced expression of M-CSF and also the increased infiltration of macrophages into tumor tissues. And it needs further investigation to reveal the underlying mechanisms.

## 4. Conclusions

In this study, we demonstrated that exogenous overexpression of ATF4 in mouse breast cancer cells can increase proangiogenic macrophages infiltrating into tumor tissues via the secretion of M-CSF, thus promoting the tumor angiogenesis and also tumor growth indirectly. These results provide interesting observations that ATF4 can affect tumor angiogenesis via the recruitment of macrophages into TME of breast cancer and also novel insights into the role of ATF4 on tumor angiogenesis besides the direct regulation of proangiogenic factors.

## Supplementary Material

To verify the efficiency of lentivirus transfection, we detected the expression level of ATF4 in 4T1-Luc-ATF4 cells and 4TO7-ATF4 cells. Both of them showed significant overexpression of ATF4 compared to corresponding control groups, as shown in Figure S1(a). Meanwhile, ATF4 overexpression did not show apparent influence on the expression level of Luc, as shown in Figure S1(b).
