# Supplementary material for: Activating Transcription Factor 4 Promotes Angiogenesis of Breast Cancer through Enhanced Macrophage Recruitment
Source: Biomed Res Int. 2015 Mar 25;2015:974615. doi: 10.1155/2015/974615 (PMC4391610; doi:10.1155/2015/974615)
Supplement: Supplementary file 1 — To verify the efficiency of lentivirus transfection, we detected the expression level of ATF4 in 4T1-Luc-ATF4 cells and 4TO7-ATF4 cells. Both of them showed significant overexpression of ATF4 compared to corresponding control groups, as shown in Figure S1(a). Meanwhile, ATF4 overexpression did not show apparent influence on the expression level of Luc, as shown in Figure S1(b). [file 974615.f1.pdf]

SUPPLEMENTARY FIGURE LEGENDS

Supplementary Figure 1. Overexpression of ATF4 in breast cancer cell lines. (A) Western blots confirmed the overexpression of ATF4 in 4T1-Luc-ATF4 cells and 4TO7-ATF4 cells. **(B)** The expression level of Luc was not influenced in ATF4-overexpressing 4T1-Luc cells.

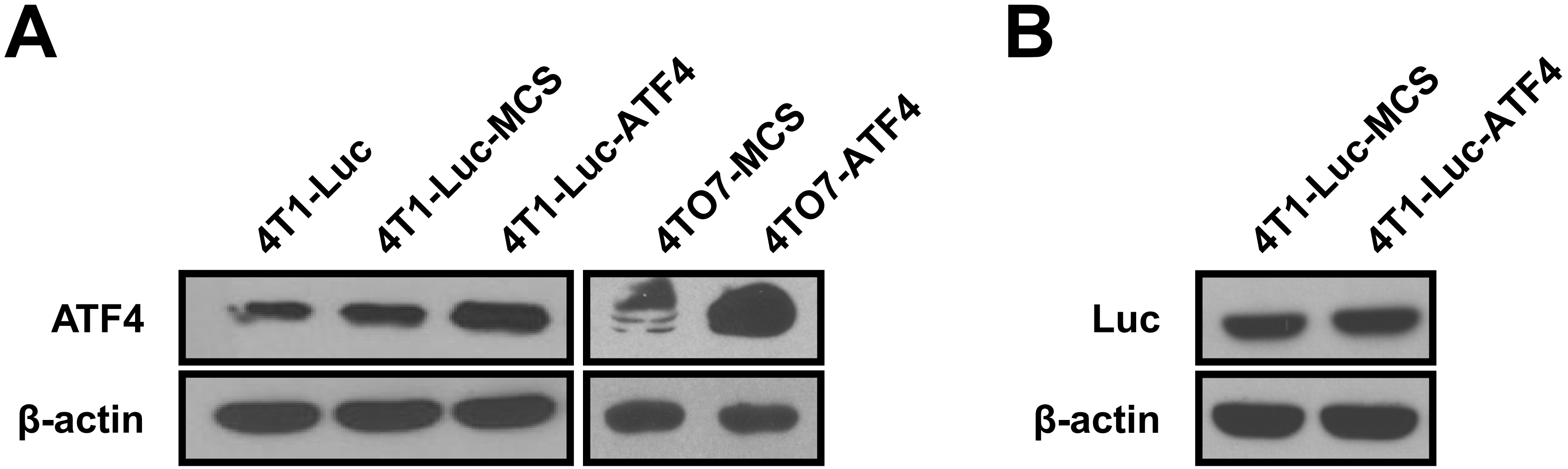

Supplementary Figure 1
